# The role of chronic conditions in influencing symptom attribution and anticipated help-seeking for potential lung cancer symptoms: a vignette-based study

**DOI:** 10.3399/bjgpopen20X101086

**Published:** 2020-08-19

**Authors:** Aradhna Kaushal, Jo Waller, Christian von Wagner, Sonja Kummer, Katriina Whitaker, Aishwarya Puri, Georgios Lyratzopoulos, Cristina Renzi

**Affiliations:** 1 Research Department of Behavioural Science and Health, University College London, London, UK; 2 School of Cancer & Pharmaceutical Sciences, King’s College London, London, UK; 3 School of Health Sciences, University of Surrey, Guildford, UK

**Keywords:** lung diseases (obstructive), lung neoplasms, general practice, primary health care, awareness, health knowledge

## Abstract

**Background:**

Very little is known about the influence of chronic conditions on symptom attribution and help-seeking for potential cancer symptoms.

**Aim:**

To determine if symptom attribution and anticipated help-seeking for potential lung cancer symptoms is influenced by pre-existing respiratory conditions (often referred to as comorbidity), such as asthma or chronic obstructive pulmonary disease (COPD).

**Design & setting:**

A total of 2143 adults (1081 with and 1062 without a respiratory condition) took part in an online vignette survey.

**Method:**

The vignette described potential lung cancer symptoms (persistent cough and breathlessness) after which questions were asked on symptom attribution and anticipated help-seeking.

**Results:**

Attribution of symptoms to cancer was similar in participants with and without respiratory conditions (21.5% and 22.1%, respectively). Participants with respiratory conditions, compared with those without, were more likely to attribute the new or changing cough and breathlessness to asthma or COPD (adjusted odds ratio [OR] = 3.64, 95% confidence interval [CI] = 3.02 to 4.39). Overall, 56.5% of participants reported intention to seek help from a GP within 3 weeks if experiencing the potential lung cancer symptoms. Having a respiratory condition increased the odds of prompt help-seeking (OR = 1.25, 95% CI = 1.04 to 1.49). Regular healthcare appointments were associated with higher odds of anticipated help-seeking.

**Conclusion:**

Only one in five participants identified persistent cough and breathlessness as potential cancer symptoms, and half said they would promptly seek help from a GP, indicating scope for promoting help-seeking for new or changing symptoms. Chronic respiratory conditions did not appear to interfere with anticipated help-seeking, which might be explained by regular appointments to manage chronic conditions.

## How this fits in

Very little is known about how pre-existing chronic conditions may influence the time between first noticing potential cancer symptoms and seeking help from a health professional. This study investigated how people with respiratory conditions would interpret and seek help for potential lung cancer symptoms. Having a chronic respiratory condition does not appear to interfere with help-seeking, despite patients attributing symptoms to their pre-existing condition. This suggests an opportunity for early cancer diagnosis during and after the first medical encounter.

## Introduction

Approximately 50% of the UK population have at least one long-standing health problem, and 25% have ≥2 conditions.^1,2^ The majority of patients diagnosed with cancer have a pre-existing condition (sometimes referred to as comorbidity); therefore, it is important to examine the potential influence of chronic conditions on the timeliness of cancer diagnoses.^3–5^ Pre-existing morbidities are associated with advanced stage or emergency cancer diagnosis, but the underlying mechanisms are not well understood.^6,7^ Some studies focused on their possible effect on the diagnostic interval (the time between the first consultation with a healthcare professional and cancer diagnosis), but relatively little is known about how chronic conditions may influence the patient interval (the time between first noticing a symptom and seeking help from a healthcare professional).^8–11^

One hypothesis is that pre-existing conditions could affect symptom attribution (that is, the perceived cause of symptoms) and help-seeking, particularly when cancer and comorbidity have overlapping symptomatology. This could lead to longer patient intervals with delays in cancer diagnoses, but the evidence is heterogeneous and dependent on the specific chronic condition and cancer site.^12–14^ In the case of lung cancer symptoms, such as persistent cough, when patients have a pre-existing respiratory condition this could provide a plausible ‘alternative explanation’, with patients underestimating the need for prompt help-seeking. Small qualitative studies^10,15,16^ and a survey retrospectively collecting information from patients with cancer^3^ suggested a similar mechanism, but recall bias might have influenced the findings. Vignette studies can be particularly useful in early diagnosis research for providing evidence on complex phenomena.^17–24^ By manipulating in a standardised way symptoms and comorbidities, and simultaneously maintaining other factors as constant (for example, healthcare and patient factors), they can provide quantitative estimates of comorbidity-specific effects and evaluate underlying mechanisms.

Population surveys have shown that 26% of the general public in England are aware that persistent cough might be a possible symptom of lung cancer.^25^ However, evidence is lacking on the impact that pre-existing conditions have on attribution of symptoms to cancer and how this might influence help-seeking.

The present study aimed to test the ‘alternative explanation hypothesis’ by using a vignette study. This was undertaken by comparing the perceived cause of the symptoms (‘symptom attribution’ hereafter) and anticipating help-seeking for potential lung cancer symptoms in people with and without pre-existing lung conditions, such as asthma or COPD. The study also aimed to evaluate whether severity and management of respiratory conditions may influence anticipated time to help-seeking for potential lung cancer symptoms, hypothesising that regular consultations would increase the likelihood of prompt help-seeking.

## Method

### Design & setting

An online cross-sectional vignette survey was carried out in June 2018 with participants recruited from the Norstat online panel.^26^ Norstat’s UK panel consists of 20 000 people (66% female) with an equal number of participants across all age groups and an average response rate of 30%. Panel members are recruited online and by phone (>50% by phone) to increase representativeness. For the survey, panel members were contacted by email and asked whether they would be interested in participating in a study on health-related issues without mentioning the word ‘cancer’. Eligibility criteria were: living in the UK, aged ≥50 years, and no history of cancer in the previous 5 years. Quota sampling ensured that 50% had a diagnosis of a respiratory condition. Sample size calculations using pilot study data indicated a sample of 2000 participants would be sufficient to detect a difference of 10% (80% power, *P*<0.05) in prompt anticipated help-seeking between participants with and without respiratory conditions.

### Vignette

All participants were shown a vignette describing new or changing symptoms of persistent cough and breathlessness over 3 weeks:

*'You have been coughing for the past 3 weeks. You also noticed that you have been short of breath when doing household chores or when climbing stairs. Aside from these changes you have not noticed anything about your health that is different from usual.'*

The choice of a cough and breathlessness was based on their high frequency as lung cancer symptoms and their high prevalence as symptoms of COPD and asthma.^27–29^ Coughing up blood was not included in the vignette to mask the cancer context of the survey, given the high public awareness of this as a symptom of lung cancer.^30^ The vignette-based survey was informed by previous research and refined using ‘think aloud’ cognitive interviews with 11 research volunteers representative of the target sample.^31,32^ To ensure that participants had understood the vignette fully, two questions were asked to test understanding of the nature and duration of symptoms. Participants had to answer these questions correctly before continuing to the next question.

### ​Measures

After reading the vignette, participants were asked a range of questions on symptom attribution, actions taken in response to new symptoms and anticipated time to help-seeking from the GP, friends and family, the pharmacy, and from accident and emergency (A&E). For participants who said they would not see their GP, barriers to anticipated help-seeking were evaluated. Details of these measures are summarised in Box 1.

Box 1 **​Details of outcome measures used in analysis**

**Symptom attribution**

After reading the vignette, participants were asked what they thought the symptoms were due to, and to write everything that came to mind. These free-text responses were analysed and coded by two researchers using content analysis to identify a range of attributions.^20^ These attributions were coded as: 'cancer', 'asthma or COPD', 'other lung disease', 'infection', 'allergies', 'heart problems', 'obesity', 'smoking', 'environment', and 'don’t know'.

**Responding to new symptoms**

*Anticipated help-seeking and other actions*

Participants were asked how they would respond if they were to experience symptoms as described in the vignette. Pre-coded answers included the following: ‘talk to friends and family’, ‘go to the pharmacy’, ‘contact the GP’, ‘mention the symptoms to the GP or nurse if seen for another reason’, ‘go to A&E’, ‘look up information online’, ‘wait and see’, ‘dismiss the symptoms as not worth worrying about’, ‘use previously prescribed medication’, or ‘use over-the-counter medication’. The order of options was randomised for each participant; response options were coded as 'would' and 'wouldn’t', with those selecting 'not applicable' excluded.

*Time to anticipated help-seeking*

Time to anticipated help-seeking was measured, with pre-coded response options including 'within 3 weeks' versus 'more than 3 weeks or not at all'. This cut-off was used to align with advice given in the Department of Health’s *Be Clear on Cancer* lung cancer awareness campaign, which has been running since 2010 and recommends seeking help after coughing for 3 weeks.^51^

*Barriers to help-seeking*

Participants selected all that applied from: 'I have other health problems that are more important', 'I don’t think the symptoms are serious', 'I’ve had the symptoms before', 'It would be a waste of my time', 'I prefer to self-manage my symptoms', 'I’ve been tested for the symptoms before', 'It would be a waste of the GP’s time', 'I don’t think the GP would be able to help', 'the GP would not take the symptom seriously', and 'I would worry about any tests I might need to have'.

*​Respiratory condition management, duration, and severity*

Participants with a respiratory condition reported whether their condition was managed in primary care and/or secondary care, and whether they were taking medications for their condition. All response options were binary ('yes' or 'no'). Further questions were asked about how long participants had their respiratory condition for (<6 months, 6–12 months, 1–5 years, and >5 years) and how frequently it limited their daily activities ('never or rarely', 'sometimes', 'very often or always').

### Covariates

All models were adjusted for the number of chronic conditions, sociodemographic variables (age, sex, ethnic group, and educational attainment) and smoking status (never, previous, or current). The presence of chronic conditions was assessed using a question adapted from the GP Patient Health Survey.^33^ As respiratory conditions were the main focus of the study, they were examined separately as a potential alternative explanation for the cough and breathlessness symptoms described in the scenario. All other conditions were summed to create a total number of other health conditions ranging from 0 to ≥3.

### Analyses

Initially, characteristics of participants were compared with and without a chronic respiratory condition using χ^2^ tests. The associations between having a respiratory condition and the following outcomes were then examined: a) symptom attribution; b) anticipated help-seeking and other actions; c) anticipated time to help-seeking; and d) reasons for not visiting the GP. Each outcome was included in a separate logistic regression model, with models a)–c) including the total study sample (*N* = 2143), while model d) only included the subgroup reporting that they would not seek help (*n* = 489). All models were adjusted for variables thought a priori to be potentially important explanatory factors based on previous evidence and clinical reasoning (that is, age, sex, education, ethnic group, marital status, smoking status, and number of prior morbidities), as well as variables associated with the outcome of interest at univariable analyses. Models b) and c) testing associations with anticipated help-seeking were also adjusted for cancer attributions, as this was considered a priori to be a potentially important explanatory variable. All regression models were tested for interactions between health conditions and sociodemographic factors. In the subgroup of participants with respiratory conditions, the study also examined the associations between duration, severity, and management of respiratory conditions with anticipated time to help-seeking using logistic regression models. The analyses were initially performed grouping together participants with asthma and/or COPD; subsequently, the study specifically focused on participants with COPD, given their higher risk of lung cancer.^34,35^

## Results

### Participants

A total of 6943 participants were screened for inclusion in the survey. After excluding ineligible participants, 2143 comprised the final analytical sample. Overall, 51.4% of participants were female and 84.2% lived in England, with smaller proportions coming from Scotland, Wales, and Northern Ireland, in line with UK population statistics; 97.0% were from a white ethnic background, which is higher compared to census data (86%).^36^ Supplementary Figure S1 shows the response and completion rate for the survey. Participants with a respiratory condition were more likely to be younger, female, to have ≥3 pre-existing conditions, and to be smokers or previous smokers compared with those without a respiratory condition (Table 1). Among participants with a respiratory condition, 688 (63.6%) had asthma, 264 (24.4%) had COPD, and 126 (11.7%) had both conditions (Table 1).

**Table 1.** Participant characteristics

Characteristics	**Total** **(*N* =** 2143), %	**With a respiratory condition** **(*n* =** 1081), %	**Without a respiratory condition** **(*n* =** 1062), %	***P* value** **(χ2 test**)
**Age, years**				
50–59	43.4	46.2	40.5	<0.05
60–69	36.8	35.3	38.2	
≥70	19.9	18.5	21.3	
**Sex**				
Male	48.6	42.8	54.4	<0.001
Female	51.4	57.2	45.6	
**Education**				
No qualifications	9.2	10.6	7.7	0.06
Secondary	46.3	46.4	46.3	
Higher education	44.5	43.1	46.0	
**Marital status**				
Single	38.5	39.0	38.1	0.7
Married or living with partner	61.5	61.1	61.9	
**Ethnic group**				
White	97.0	97.2	96.8	0.6
Other	3.2	2.8	3.2	
**Region**				
England	84.2	83.2	85.3	0.2
Scotland	8.7	9.9	7.4	
Wales	4.8	4.8	4.7	
Northern Ireland	2.3	2.1	2.5	
**Smoking**				
Current smoker	18.3	22.0	14.5	<0.001
Previous smoker	40.7	42.8	38.5	
Never smoked	41.0	35.2	47.0	
**Other health conditions, *n***				
0	22.9	18.4	27.5	<0.001
1	21.4	19.9	22.9	
2	20.0	19.2	20.7	
≥3	35.7	42.5	28.9	
**Type of respiratory condition**				
Asthma		63.6		
COPD		24.4		
COPD and asthma		11.7		
**Primary care management for respiratory condition**				
Yes		81.7		
No		18.3		
**Secondary care management for respiratory condition**				
Yes		14.8		
No		85.2		
**Over-the-counter medication taken for respiratory condition**				
Yes		3.0		
No		97.0		
**Respiratory condition limits daily activities**				
Never or rarely		47.2		
Sometimes		30.9		
Very often or always		21.9		
**Duration of respiratory condition, years**				
≤5		25.9		
>5		74.1		

COPD = chronic obstructive pulmonary disease.

### Symptom attribution

There was a similarity between participants with and without a respiratory condition in attributing symptoms to cancer (21.5% and 22.1%, respectively). Participants with a chronic respiratory condition most frequently attributed the symptoms described in the vignette to asthma or COPD (62.6%); while individuals without respiratory conditions most frequently attributed symptoms to an infection (38.9%). Half of participants, independently of chronic condition status, mentioned only one symptom attribution; multiple attributions were more frequently reported by participants with chronic conditions (Table 2).

**Table 2.** Symptom attribution among participants with and without respiratory conditions

	**Without a respiratory condition (*n* =** 1062), %	**With a respiratory condition (*n* =** 1081), %	**Total (*N* =** 2143), %	***P* value (χ2 test**)
**Symptom attribution**				
Cancer	22.1	21.5	21.8	0.709
Asthma or COPD	30.8	62.6	46.9	<0.001
Other lung disease	10.9	7.0	9.0	0.002
Infection	38.9	35.2	37.0	0.073
Allergies	8.2	12.5	10.4	0.001
Heart problems	15.1	10.6	12.8	0.002
Obesity	3.0	2.5	2.8	0.466
Smoking	3.4	3.7	3.6	0.698
Environment	1.4	3.5	2.5	0.002
Don’t know	14.4	4.0	9.2	<0.001
**Symptom attributions, *n***				
0	18.0	5.9	11.9	<0.001
1	47.9	49.6	48.8	
2	20.7	28.2	24.5	
≥3	13.4	16.3	14.8	

COPD = chronic obstructive pulmonary disease.

**Figure 1.**
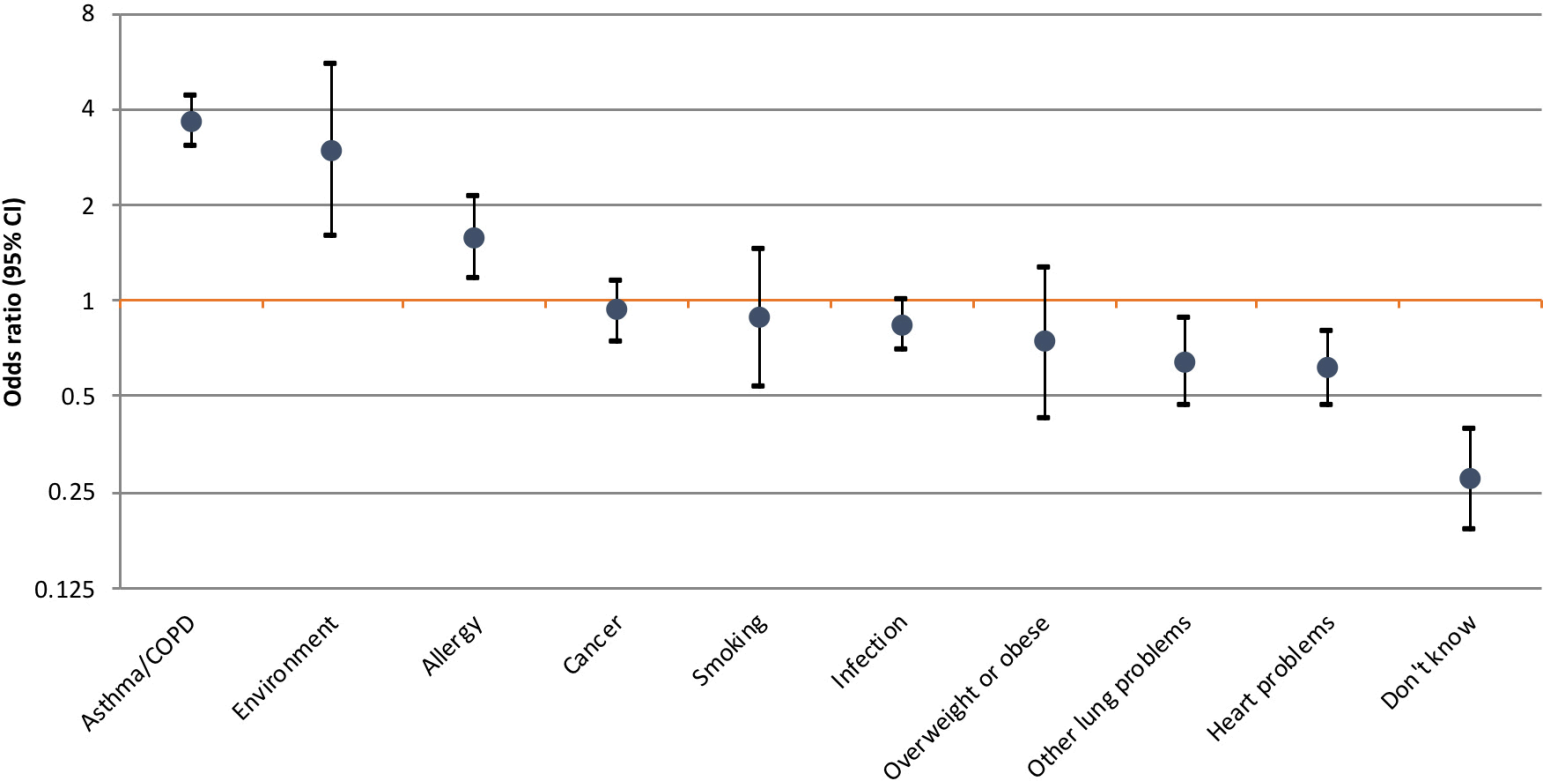
Odds ratios for different symptom attributions for participants with respiratory conditions compared with participants without respiratory conditions (adjusted for other health conditions, sex, age, education, smoking status, and marital status; *n* = 2143). Each estimate is derived by a different model with the condition of interest as a yes or no outcome variable. COPD = chronic obstructive pulmonary disease

Figure 1 shows that having a respiratory condition was associated with higher odds of attributing symptoms to the pre-existing condition (OR = 3.64, 95% CI = 3.02 to 4.39), a change in the environment (OR = 2.95, 95% CI = 1.59 to 5.49), and allergies (OR = 1.57, 95% CI = 1.16 to 2.10), taking other health conditions, smoking status, and sociodemographic characteristics into account. Individuals with a respiratory condition were less likely to attribute symptoms to other lung problems (OR = 0.64, 95% CI = 0.47 to 0.87), heart problems (OR = 0.61, 95% CI = 0.47 to 0.80), or to say they did not know what the symptom could be (OR = 0.27, 95% CI = 0.19 to 0.39). Further details are reported in Supplementary Table S1.

Focusing specifically on participants with COPD showed similar findings to those observed for the combination of COPD and/or asthma. Cancer attribution was reported by 19.5% of participants with COPD; in multivariable analyses COPD increased the odds of attributing symptoms to the pre-existing condition (OR = 3.50, 95% CI = 2.67 to 4.57) and a change in the environment (OR = 4.95, 95% CI = 2.10 to 11.68), with no effect on cancer attribution (OR = 0.82, 95% CI = 0.60 to 1.12).

### Responding to new symptoms

#### Anticipated help-seeking and other actions

Participants indicated a median number of five possible actions (interquartile range [IQR] 4–7 actions), with no difference by chronic disease status. The most frequently reported action was ‘mention the symptoms to the GP or nurse if they saw them for another reason’ (89.7%), followed by ‘contact the GP’ (76.7%), ‘talk to friends or family’ (64.1%), and ‘look up information online’ (63.8%). Over a quarter (28.3%, *n* = 601) said they would ‘dismiss the symptoms as not worth worrying about’. The majority of this subgroup, while not worrying about the symptoms, would still ‘mention the symptoms to the GP if seen for another reason’ (86.7%, *n* = 521/601), with no difference by chronic disease status. At multivariable analyses there were no differences in intention to contact the GP between participants with and without a respiratory condition (OR = 1.08, 95% CI = 0.88 to 1.34; 77.3% versus 76.1%; *n* = 824 versus *n* = 785; Table 3). Participants with a respiratory condition were less likely to say they would ‘go to the pharmacy’ for advice (OR = 0.70, 95% CI = 0.58 to 0.84; 49.5% versus 58.3%), ‘use over-the-counter medicine’ (OR = 0.68, 95% CI = 0.56 to 0.81; 35.8% versus 44.8%), ‘look up information online’ (OR = 0.79, 95% CI = 0.66 to 0.96; 61.3% versus 66.4%), or ‘dismiss the symptoms as not worth worrying about’ (OR = 0.76, 95% CI = 0.62 to 0.93; 26.3% versus 30.5%).

**Table 3.** Anticipated actions (probably or definitely would) in response to new symptoms by chronic respiratory condition status, sociodemographic factors, and cancer symptom attribution: multivariable logistic regression analyses

	**Contact the GP (*n* = 2098**)	**Go to the** **pharmacy**(***n* = 2105**)	**Go to A&E**(***n* = 2093**)	**Mention the** **symptoms to the GP if seen for another reason**(***n* = 2094**)	**Use previously prescribed** **medication (*n* = 1411**)	**Use over-the-counter** **medication (*n* = 2089**)	**Look up** **information online**(***n* = 2095**)	**Wait and see**(***n* = 2112**)	**Dismiss the symptoms as not worth worrying about (*n* = 2121**)
	**OR (95% CI**)	**OR (95% CI**)	**OR (95% CI**)	**OR (95% CI**)	**OR (95% CI**)	**OR (95% CI**)	**OR (95% CI**)	**OR (95% CI**)	**OR (95% CI**)
**Unadjusted**										
**Respiratory condition**										
Yes	1.07 (0.88 to 1.31)	0.70 (0.59 to 0.83)^a^	1.36 (0.99 to 1.89)	1.28 (0.96 to 1.69)	5.46 (4.3 to 6.9)^a^	0.69 (0.58 to 0.82)^a^	0.80 (0.67 to 0.95)^b^	0.88 (0.74 to 1.04)	0.81 (0.67 to 0.98)^b^	
No	1.0	1.0	1.0	1.0	1.0	1.0	1.0	1.0	1.0	
**Adjusted**										
**Respiratory condition**										
Yes	1.08 (0.88 to 1.34)	0.70 (0.58 to 0.84)^a^	1.27 (0.91 to 1.79)	1.23 (0.92 to 1.65)	5.34 (4.19 to 6.80)^a^	0.68 (0.56 to 0.81)^a^	0.79 (0.66 to 0.96)^b^	0.84 (0.70 to 1.01)	0.76 (0.62 to 0.93)^c^	
No	1.0	1.0	1.0	1.0	1.0	1.0	1.0	1.0	1.0	
**Other health conditions, *n***										
0	1.0	1.0	1.0	1.0	1.0	1.0	1.0	1.0	1.0	
1	1.11 (0.82 to 1.50)	1.20 (0.92 to 1.56)	1.00 (0.60 to 1.67)	1.24 (0.83 to 1.86)	1.09 (0.76 to 1.56)	1.03 (0.79 to 1.34)	1.27 (0.96 to 1.67)	0.92 (0.70 to 1.20)	0.99 (0.74 to 1.31)	
2	1.03 (0.75 to 1.40)	1.17 (0.89 to 1.52)	0.87 (0.51 to 1.47)	1.26 (0.84 to 1.91)	0.93 (0.65 to 1.34)	0.78 (0.59 to 1.03)	1.20 (0.90 to 1.58)	0.88 (0.67 to 1.15)	0.89 (0.66 to 1.20)	
≥3	1.26 (0.96 to 1.67)	1.16 (0.92 to 1.47)	1.25 (0.80 to 1.95)	1.83 (1.25 to 2.69)^c^	1.30 (0.94 to 1.79)	1.00 (0.79 to 1.28)	0.97 (0.76 to 1.24)	0.94 (0.74 to 1.20)	0.98 (0.76 to 1.27)	
**Age, years**										
50–59	1.0	1.0	1.0	1.0	1.0	1.0	1.0	1.0	1.0	
60–69	1.30 (1.03 to 1.64)^b^	0.83 (0.68 to 1.02)	0.76 (0.52 to 1.09)	1.57 (1.12 to 2.20)^c^	0.90 (0.69 to 1.18)	0.74 (0.60 to 0.90)^c^	0.75 (0.61 to 0.93)^c^	0.82 (0.66 to 1.00)^b^	0.85 (0.68 to 1.06)	
≥70	1.52 (1.13 to 2.05)^c^	1.12 (0.87 to 1.43)	0.43 (0.25 to 0.75)^c^	1.23 (0.83 to 1.82)	0.68 (0.49 to 0.95)^b^	0.65 (0.50 to 0.83)^c^	0.58 (0.45 to 0.75)^a^	0.63 (0.49 to 0.81)^a^	0.50 (0.38 to 0.67)^a^	
**Sex**										
Male	1.0	1.0	1.0	1.0	1.0	1.0	1.0	1.0	1.0	
Female	0.88 (0.72 to 1.09)	1.10 (0.92 to 1.32)	0.72 (0.51 to 1.01)	1.33 (0.99 to 1.78)	1.12 (0.88 to 1.43)	1.02 (0.85 to 1.23)	1.25 (1.04 to 1.51)^b^	1.37 (1.15 to 1.65)^c^	1.14 (0.93 to 1.39)	
**Education**										
Higher education	1.0	1.0	1.0	1.0	1.0	1.0	1.0	1.0	1.0	
No qualifications	1.21 (0.81 to 1.80)	0.98 (0.71 to 1.35)	2.61 (1.57 to 4.33)^a^	0.69 (0.42 to 1.15)	0.77 (0.50 to 1.17)	1.02 (0.73 to 1.43)	0.60 (0.43 to 0.83)^b^	0.99 (0.72 to 1.37)	1.39 (0.98 to 1.98)	
Secondary education	1.07 (0.87 to 1.33)	1.23 (1.03 to 1.48)^b^	1.15 (0.80 to 1.65)	0.85 (0.62 to 1.15)	0.89 (0.69 to 1.15)	1.06 (0.88 to 1.28)	0.91 (0.75 to 1.11)	0.91 (0.76 to 1.10)	1.20 (0.98 to 1.47)	
**Ethnic group**										
White	1.0	1.0	1.0	1.0	1.0	1.0	1.0	1.0	1.0	
Non-white	2.63 (1.23 to 5.63)	2.05 (1.15 to 3.65)^b^	5.99 (3.28 to 10.95)^a^	1.77 (0.63 to 5.01)^b^	2.22 (1.04 to 4.72)^b^	2.26 (1.31 to 3.90)^c^	1.96 (1.03 to 3.75)	0.73 (0.43 to 1.24)	1.20 (0.70 to 2.07)	
**Marital status**										
Single	1.0	1.0	1.0	1.0	1.0	1.0	1.0	1.0	1.0	
Married or living with partner	0.94 (0.76 to 1.16)	1.04 (0.87 to 1.25)	0.79 (0.57 to 1.11)	1.01 (0.75 to 1.35)	1.14 (0.89 to 1.46)	1.09 (0.90 to 1.31)	1.11 (0.92 to 1.34)	1.15 (0.96 to 1.39)	1.02 (0.84 to 1.25)	
**Smoking status**										
Never smoked	1.0	1.0	1.0	1.0	1.0	1.0	1.0	1.0	1.0	
Current smoker	0.85 (0.64 to 1.13)	0.82 (0.64 to 1.06)	1.40 (0.91 to 2.15)	0.68 (0.46 to 1.00)	1.44 (1.03 to 2.02)^b^	0.92 (0.71 to 1.19)	0.78 (0.60 to 1.01)	0.87 (0.67 to 1.12)	1.20 (0.91 to 1.57)	
Previous smoker	1.15 (0.90 to 1.45)	0.78 (0.64 to 0.95)^b^	0.97 (0.65 to 1.43)	0.87 (0.62 to 1.21)	0.94 (0.72 to 1.23)	0.88 (0.72 to 1.08)	1.09 (0.88 to 1.34)	0.82 (0.67 to 1.01)	0.97 (0.78 to 1.21)	
**Symptom attribution**										
Cancer	1.50 (1.15 to 1.95)^c^	0.79 (0.64 to 0.97)^b^	0.69 (0.44 to 1.08)	1.25 (0.87 to 1.80)	0.83 (0.62 to 1.11)	0.85 (0.68 to 1.06)	1.24 (0.99 to 1.55)	0.71 (0.58 to 0.88)^c^	0.61 (0.48 to 0.79)^a^	

^a^<0.001. ^b^<0.05. ^c^<0.01. OR = odds ratio.

#### Time to anticipated help-seeking from the GP

Examining time to seeking help from the GP has shown that 56.5% of participants with a respiratory condition (62.5% specifically among participants with COPD) would seek help within 3 weeks, compared with 54.2% of those without respiratory conditions.

At multivariable analyses, having a respiratory condition increased the odds of anticipated help-seeking within 3 weeks (OR = 1.25, 95% CI = 1.04 to 1.49), controlling for sociodemographic factors, smoking, and cancer symptom attribution. Similar findings were observed when focusing on individuals with COPD (Table 4).

**Table 4.** Seeking help from the GP within 3 weeks for potential lung cancer symptoms by chronic respiratory conditions, sociodemographic factors, and symptom attribution: multivariable logistic regression analyses

	**Individuals with COPD and/or asthma versus no respiratory conditions** **(*n* = 2084**), **OR (95% CI**)	**Individuals with COPD versus no respiratory condition** **(*n* = 1402**), **OR (95% CI**)
**Unadjusted**		
**Respiratory condition**		
No	1.0	1.0
Yes	1.20 (1.01 to 1.42)^a^	1.41 (1.11 to 1.79)^b^
**Adjusted**		
**Respiratory condition**		
No	1.0	1.0
Yes	1.25 (1.04 to 1.49)^a^	1.36 (1.04 to 1.78)^a^
**Other health conditions, *n***		
0	1.0	1.0
1	0.98 (0.75 to 1.27)	0.89 (0.65 to 1.23)
2	1.02 (0.78 to 1.34)	1.08 (0.78 to 1.50)
≥3	1.20 (0.94 to 1.53)	1.36 (1.01 to 1.83)^a^
**Age, years**		
50–59	1.0	1.0
60–69	1.13 (0.92 to 1.38)	1.19 (0.93 to 1.54)
≥70	1.29 (1.00 to 1.65)^a^	1.32 (0.98 to 1.77)
**Sex**		
Male	1.0	1.0
Female	0.59 (0.49 to 0.71)^c^	0.51 (0.41 to 0.64)^c^
**Education**		
Higher education	1.0	1.0
No qualifications	1.08 (0.77 to 1.50)	1.04 (0.70 to 1.54)
Secondary education	0.94 (0.78 to 1.13)	0.92 (0.73 to 1.16)
**Ethnic group**		
White	1.0	1.0
Non-white	2.34 (1.32 to 4.14)^b^	2.27 (1.07 to 4.08)^a^
**Marital status**		
Single	1.0	1.0
Married or living with partner	0.93 (0.77 to 1.11)	0.99 (0.79 to 1.23)
**Smoking status**		
Never smoked	1.0	1.0
Current smoker	1.04 (0.81 to 1.34)	1.02 (0.74 to 1.41)
Previous smoker	1.13 (0.93 to 1.39)	1.07 (0.83 to 1.39)
**Symptom attribution**		
Cancer	1.08 (0.87 to 1.34)	1.11 (0.85 to 1.45)

^a^<0.05. ^b^<0.01. ^c^<0.001. OR = odds ratio.

Multivariable analysis has also shown that the likelihood of prompt anticipated help-seeking was higher for older aged participants, while it was lower for females. Examining whether sex modified the effect of chronic respiratory conditions on help-seeking yielded no strong evidence for interaction (likelihood ratio test for interaction between sex and chronic respiratory condition was *P* = 0.065 for individuals with COPD and/or asthma, and *P* = 0.689 for the analyses focusing on COPD only).

#### Anticipated barriers to help-seeking

**Figure 2.**
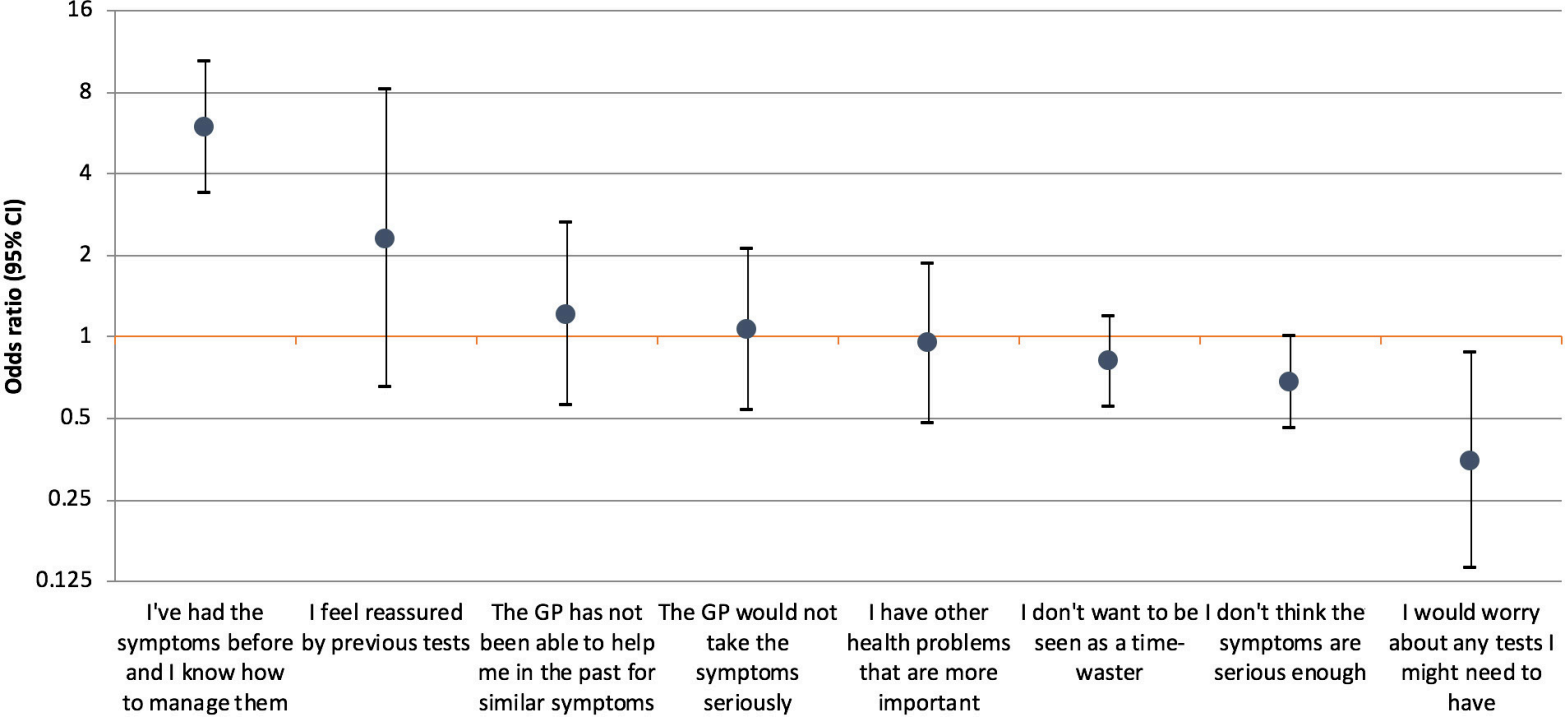
Odds ratios for reasons for not contacting the GP for participants with respiratory conditions compared with participants without respiratory conditions (adjusted for other health conditions, sex, age, education, smoking status, and marital status; *n* = 489). Each estimate is derived by a different model with the condition of interest as a yes or no outcome variable

Among participants who said they would not contact their GP (*n* = 489), those with a respiratory condition were more likely to say that they had experienced the symptoms before and knew how to manage them (OR = 5.30, 95% CI = 3.11 to 9.01; 31.8% versus 8.1%), and were less likely to say they would worry about any tests they might need to have (OR = 0.35, 95% CI = 0.14 to 0.87; 3.3% versus 7.3%; Figure 2). Further details are reported in Supplementary Table S2.

#### Management, duration, and severity of respiratory conditions

**Figure 3.**
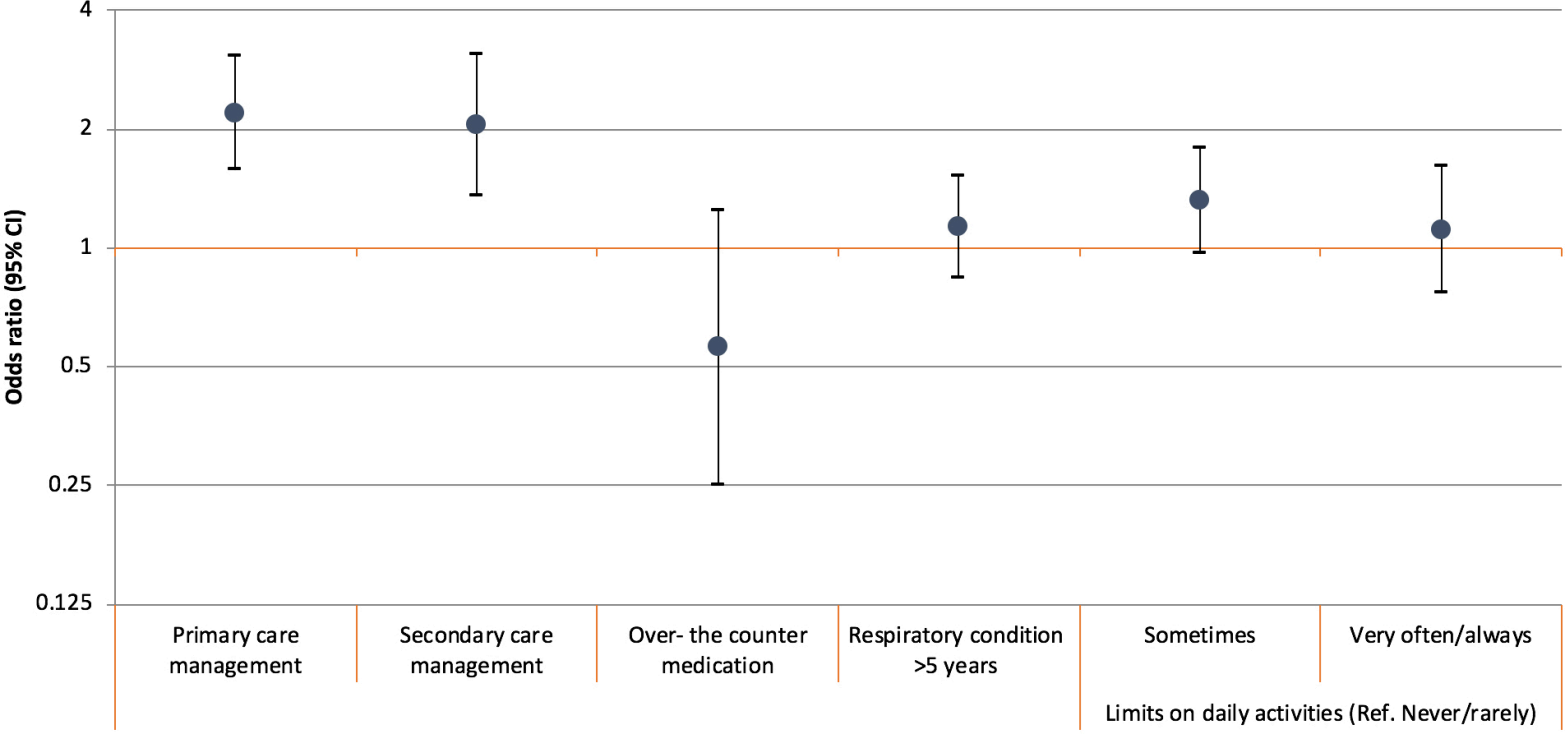
Associations between respiratory condition management, duration and limits on daily activity, and help-seeking within 3 weeks among participants with a respiratory condition, *n* = 1081

Figure 3 shows how the management, duration, and severity of respiratory conditions were associated with help-seeking from the GP within 3 weeks. Having regular appointments in primary and secondary care for respiratory conditions was associated with higher odds of help-seeking from the GP (OR = 2.20, 95% CI = 1.58 to 3.06, 86.61% versus 74.3%; OR = 2.05, 95% CI = 1.36 to 3.09, 18.7% versus 9.4%, respectively). Participants whose respiratory condition sometimes limited their daily activities had somewhat higher odds of prompt help-seeking compared with those with less severe conditions (never or rarely limiting their activities), but not at statistically significant levels (OR = 1.32, 95% CI = 0.97 to 1.80).

## Discussion

### Summary

Overall, one in five participants attributed new or changing symptoms of persistent cough and breathlessness to cancer, with no difference found between participants with and without a respiratory condition. People with a respiratory condition were more likely to attribute the persistent cough and breathlessness to the pre-existing condition, consistent with the 'alternative explanation' hypothesis, and were more likely to anticipate using previously prescribed medications to treat the symptoms. Overall, one in two participants reported they would seek help promptly if they were to experience the symptoms described in the vignette. Contrary to expectations, there was no association between presence of a lung comorbidity and prolonging the time before seeking help from the GP. There was some evidence that having a chronic respiratory condition may increase the likelihood of prompt help-seeking for new symptoms, particularly for individuals whose chronic conditions are regularly managed in primary and secondary care.

### Strengths and limitations

The vignette design allowed the authors to perform an in-depth examination of the possible mechanism linking pre-existing chronic conditions to symptom attribution and help-seeking on a large sample of participants from the four nations of the UK. A strength of the vignette methodology is that it is able to present a standardised realistic scenario controlling for other variables. Symptom and chronic disease severity could be accounted for in this study, which can influence symptom attribution and help-seeking.^37^ The survey completion rate was very high, with negligible missing data. It is possible that people who are more likely to seek help from the GP are over-represented among survey participants, potentially leading to an overestimation of help-seeking behaviour.

By the very nature of the study, the authors were only able to ask about anticipated help-seeking in relation to a hypothetical scenario. Although intentions do not always translate into actual help-seeking, there is clear evidence that formulating intentions is a vital step in the process to taking action.^38,39^

It is not possible to account for the effect of all factors playing a role in the decision to seek help (for example, progression of symptoms, response to medications) using a vignette design.^40^ Owing to the relatively low prevalence of COPD in the sample, participants with asthma and COPD were initially combined, but additional analyses focusing on the relatively small subgroup of participants with COPD showed similar findings.

Owing to multiple testing, the possibility of some false positive associations cannot be excluded. However, as pre-defined hypotheses were tested this might be a less relevant issue than in other types of studies, such as genomic research. Moreover, while different approaches have been proposed (for example, Bonferroni) for correcting for multiple testing, each has its limitations and there is no overall agreement in the literature on how to deal with this issue.^41^ Some authors suggest that multiple-test corrections should not be used in scientific experiments to avoid missing possibly important findings.^42^

### Comparison with existing literature

The low level of lung cancer symptom attribution found in this study is in line with previous reports on lung cancer awareness in the UK.^30,43^ Cancer symptom awareness has been reported to be similar in the UK compared with other countries.^44^ The finding in the present study that around half of people would seek help promptly for cough and breathlessness from the GP is in agreement with previous research.^11^ Similar to previous qualitative studies with patients with lung cancer,^10,15,45^ it was found that respiratory comorbidity can influence symptom attribution owing to overlapping symptomatology. However, no difference was found between those with and without respiratory conditions in attributing the symptoms to cancer.

The finding that participants with a respiratory condition, compared with those without, have higher odds of anticipated prompt help-seeking contradicts the only comparable other quantitative research, which showed that a history of COPD was associated with taking twice as long to seek help.^3^ Studies based on retrospectively asking patients with cancer might, however, be influenced by recall bias. Research in this area is lacking but it is becoming clear that comorbidities can sometimes facilitate and other times interfere with help-seeking, depending on symptom and comorbidity characteristics and previous healthcare experiences.^14^ Intention to seek help was higher among those who have regular appointments in primary and secondary care. Regular appointments can increase a patient’s familiarity with healthcare services, which may in turn facilitate reporting of new symptoms.^46^ Moreover, patients might feel that help-seeking for vague symptoms is more acceptable if the consultation is also needed for ongoing management of a comorbidity.^47^ It is also possible that positive expectations can arise from successful management of the pre-existing chronic condition encouraging prompt help-seeking.^10^

The lower likelihood of anticipated prompt help-seeking from the GP observed in the study among women contrasts with prior literature^47^ and merits further examination in subsequent studies. It might reflect differences by sex in symptom attribution and attitudes regarding symptom management.^48^ Women might have the perception that they are able to manage the symptoms without seeing the GP. While no sex difference was found in barriers to help-seeking or in cancer attribution (data not shown), women attributed symptoms more frequently to an infection compared with men (40.1% versus 33.7%, *P* = 0.002) and they more frequently reported that they would 'wait and see' and ‘look up information online’.

### Implications for research and practice

The present study findings suggest that the awareness of possible lung cancer symptoms is low, even among patients at higher risk of lung cancer, such as those with COPD.^49,50^ Mass-media campaigns offer an opportunity to address low awareness of lung cancer symptoms, with some studies having reported a trend towards earlier stage at diagnosis of lung cancer in areas targeted by cancer awareness campaigns.^25,51,52^ As half of the participants in the present study did not anticipate prompt help-seeking from the GP if they were to experience potential lung cancer symptoms, information campaigns should also emphasise the importance of promptly discussing new or changing symptoms with the doctor. A few studies have evaluated interventions for increasing prompt help-seeking for possible lung cancer symptoms, showing some benefits for patients at increased cancer risk.^53,54^ However, there are some concerns that mass-media campaigns are short-lived and may create an increased workload for primary care.^55,56^

In addition to examining respiratory conditions, further research is needed on the possible effects of other chronic diseases (for example, mental health issues) on help-seeking for potential cancer symptoms. Mediators between comorbidities and help-seeking should also be investigated, including the experience of previous doctor–patient interactions, patient priorities, overall health status, and constructs such as self-efficacy, body vigilance, and cancer worry.^10,57,58^

In order to seize the opportunities to diagnose cancer early when individuals with pre-existing conditions present to their GP with possible cancer symptoms, attention should also be dedicated to events occurring post-presentation.^59,60^ Allowing enough time to discuss all health concerns when patients have multiple conditions, and improving timely access to specialist consultations and investigations may contribute to reducing the risk of diagnostic delays and advanced cancer stage at diagnosis for these complex patients. Future efforts should also focus on improving effective safety-netting strategies for patients with multiple health conditions.^61^

Overall, only one in five individuals identified cough and breathlessness as potential cancer symptoms, indicating scope for promoting help-seeking for new or changing symptoms. Having a chronic respiratory condition does not appear to interfere with help-seeking, despite patients attributing symptoms to their pre-existing condition. As overall half of participants would not seek help in a timely fashion, information campaigns need to emphasise the importance of promptly discussing new or changing symptoms with a doctor. This is particularly relevant for people with chronic conditions, who are at increased risk of advanced stage cancer diagnosis.
